# Adjunctive short- and long-term combination treatment of esketamine and VNS in difficult to treat depression (DTD)

**DOI:** 10.1192/j.eurpsy.2023.572

**Published:** 2023-07-19

**Authors:** E. Kavakbasi, B. T. Baune

**Affiliations:** Psychiatry, University Hospital Münster, Münster, Germany

## Abstract

**Introduction:**

NMDA-Receptor antagonists have rapid antidepressant and antisuicidal properties. However, the antidepressant effect is short lasting raising the question of best maintenance strategy, which is unanswered so far. Invasive vagus nerve stimulation (VNS) as a treatment option for refractory and chronic major depression was shown to reduce the need of maintenance treatment sessions in electroconvulsive therapy (ECT) patients.

**Objectives:**

There are no published data on the combination of VNS and esketamine. To determine the impact of the combination of VNS and esketamine in DTD.

**Methods:**

In this naturalistic observational study, we investigated the short- and long-term impact of combination of VNS and esketamine in n=8 patients with difficult-to-treat depression (DTD). Follow-up evaluations were scheduled prospectively pre-surgery at baseline and every 3 months after VNS-implantation (follow-up period 12-24 months, mean 17).

**Results:**

The mean age of patients was 50,8 years. 50 % of patients (n=4) were female. All patients suffered from severe DTD (mean MADRS at baseline 30,9). Mean number of hospitalizations per months decreased from 0.17 to 0.11 after VNS implantation. 6 of 8 patients were offered maintenance esketamine treatment. Mean MADRS at 12-months was 19 (38.5 % MADRS reduction). The need of mean esketamine treatment sessions decreased from 2.3 at 6-months visit (V6) to 1.37 at V9 and 0.96 at V12 respectively. Termination of maintenance esketamine was possible in 4 cases after a mean of 11.5 months.

**Conclusions:**

Combination of esketamine and VNS is a safe and effective treatment option in severely ill DTD patients to relieve disease severity and reduce hospitalizations. Need of esketamine treatment sessions decreases 6 months after VNS implantation.

**Disclosure of Interest:**

None Declared

